# Early recurrence after pulsed field ablation: a prognostic signal that challenges, but does not eliminate, the blanking period

**DOI:** 10.1093/europace/euag100

**Published:** 2026-07-22

**Authors:** Paschalis Karakasis

**Affiliations:** Second Department of Cardiology, Medical School, Aristotle University of Thessaloniki, General Hospital Hippokration, Konstantinoupoleos 49, Thessaloniki 54642, Greece

We read with great interest the study by Rodriguez-Riascos *et al*.^1^ evaluating the prognostic significance of early recurrence after pulsed field ablation (PFA) in a large real-world cohort of 1162 patients. The authors are to be commended for addressing a clinically important and increasingly debated issue in contemporary atrial fibrillation (AF) ablation. Their findings provide important insight into the temporal significance of post-PFA atrial arrhythmias and meaningfully advance the discussion regarding the relevance of the traditional blanking period.

The central observation is compelling: early recurrence at 30, 60, and 90 days was strongly associated with subsequent late AF recurrence, with progressively greater risk across later thresholds. Moreover, in the subgroup undergoing continuous rhythm monitoring, early recurrence identified patients at substantially higher risk of clinically relevant arrhythmia burden and repeat ablation at 1 year. These findings challenge the conventional notion that early post-ablation arrhythmias are predominantly benign and transient, and instead suggest that, after PFA, such events may more often represent a marker of procedural failure or persistent arrhythmogenic substrate.^2–5^

Nevertheless, although this study strongly supports reappraisal of the clinical meaning of early recurrence after PFA, we do not believe it justifies abandonment of the blanking period construct altogether.

First, rhythm surveillance was not uniform. Patients undergoing continuous or near-continuous monitoring had substantially higher rates of detected early recurrence than those followed with conventional intermittent assessment. This is an important methodological issue, because a binary classification of early recurrence may partly reflect monitoring intensity rather than pure biological behaviour. Before redefining procedural endpoints, the field requires standardized surveillance strategies.

Second, mechanistic interpretation is limited by lesion-set heterogeneity. Nearly half of the cohort underwent ablation beyond pulmonary vein isolation, including cavotricuspid isthmus ablation, posterior wall ablation, roof lines, and mitral isthmus ablation, while adjunctive lesions were delivered using either PFA or thermal energy. Accordingly, the observed association between early and late recurrence cannot be assumed to represent a uniform PFA-specific healing profile. Rather, it likely reflects a complex interaction among energy source, lesion set, and underlying atrial substrate.

Third, the provocative suggestion that early recurrence should be approached similarly to late recurrence is clinically ahead of the available evidence. Prediction of failure is not equivalent to proof of failed lesion durability, nor does it establish that earlier reintervention improves outcomes. Even the 2024 EHRA/HRS/APHRS/LAHRS consensus moved towards shortening the blanking period to 8 weeks, not eliminating the concept altogether.^6^

In our view, the most appropriate interpretation is not that the blanking period has become obsolete after PFA, but that its traditional meaning requires refinement. Rather than a rigid 90-day convention inherited from the thermal era, the early post-PFA period may be better conceptualized as a modality-specific risk-stratification window. Future prospective studies with standardized continuous monitoring, arrhythmia-pattern adjudication, and lesion-set stratification are needed to determine whether the timing, burden, and pattern of early recurrence should redefine procedural success after PFA.
